# Editorial: Immune cell exhaustion: new challenges and opportunities in cancer therapy

**DOI:** 10.3389/fimmu.2024.1527428

**Published:** 2024-12-02

**Authors:** Hao Chi, Lai Jiang, Xuancheng Zhou, Lexin Wang, Guanhu Yang, Honghao Luo, Xiaoli Liu, Ke Xu, Gang Tian

**Affiliations:** ^1^ Clinical Medical College, Southwest Medical University, Luzhou, China; ^2^ Western(Chongqing) Collaborative, Innovation, Center for Intelligent Diagnostics and Digital Medicine, Chongqing, China; ^3^ Research Department, Swiss University of Traditional Chinese Medicine, Bad Zurzach, Switzerland; ^4^ Department of Specialty Medicine, Ohio University, Athens, OH, United States; ^5^ Department of Radiology, Xichong People’s Hospital, Nanchong, China; ^6^ Department of Gastroenterology, Xichong People’s Hospital, Nanchong, China; ^7^ Department of Oncology, Chongqing General Hospital, Chongqing University, Chongqing, China; ^8^ Department of Clinical Laboratory Medicine, The Affiliated Hospital of Southwest Medical University, Luzhou, China; ^9^ Sichuan Province Engineering Technology Research Center of Molecular Diagnosis of Clinical Diseases, Luzhou, China; ^10^ Molecular Diagnosis of Clinical Diseases Key Laboratory of Luzhou, Sichuan, China

**Keywords:** immune cell exhaustion, immunotherapy, tumor microenvironment, single-cell sequencing (scRNA-seq), therapeutic targets

## 1 Introduction

In recent years, immunotherapy has made significant progress in cancer treatment, especially in malignant tumors that are difficult to cure with traditional therapies, showing unprecedented potential (1). However, many patients do not exhibit durable responses to immunotherapy in clinical practice, and the issue of drug resistance remains prominent, making immune cell exhaustion one of the main barriers to current cancer immunotherapy (2). Immune cell exhaustion is a complex process involving prolonged antigen stimulation, activation of immunosuppressive signals, and cellular metabolic dysregulation, ultimately leading to the dysfunction of effector T cells and other immune cells, thereby weakening the anti-tumor immune response (3). Several articles in this Research Topic explore the mechanisms and countermeasures of immune cell exhaustion from innovative perspectives. The studies focus on how immunosuppressive factors and signaling pathways lead to immune cell dysfunction in the tumor microenvironment and demonstrate the application of technologies such as single-cell sequencing in revealing the dynamic changes of immune cells. Furthermore, novel therapeutic strategies combining nanotechnology and image-guided approaches are proposed, offering new ideas for reversing immune cell exhaustion and improving the effectiveness of immunotherapy.

These research findings not only deepen our understanding of the process of immune cell exhaustion but also provide potential clinical directions for the future development of personalized immunotherapy, driving the field of immunotherapy towards more precise and efficient goals.

## 2 Immune cell exhaustion and its manifestations in the tumor microenvironment

In recent years, immune cell exhaustion has been identified as one of the major obstacles in cancer immunotherapy. In the tumor microenvironment (TME), prolonged antigen stimulation and immunosuppressive signals lead to the gradual exhaustion of various immune cells, such as effector T cells and macrophages, thereby weakening their anti-tumor activity. This process is often accompanied by the high expression of inhibitory receptors (such as PD-1 and CTLA-4) and metabolic dysregulation, which further enhances the tumor’s ability to evade immune responses. Therefore, understanding the mechanisms of immune exhaustion and exploring ways to improve the TME have become crucial research areas (4).

Against this backdrop, Li et al. conducted an in-depth analysis of the role of the TUBA1C gene in clear cell renal cell carcinoma (ccRCC) and found that it activates the PI3K/AKT signaling pathway, recruits immunosuppressive cells (such as MDSCs and Tregs), and induces dysfunction of CD8+ T cells. This mechanism reveals how TUBA1C remodels the tumor microenvironment to make it more immunosuppressive, thereby contributing to resistance to immune checkpoint blockade (ICB) therapy and providing a potential target for precision treatment in ccRCC. Additionally, Wang et al. proposed a treatment strategy for pancreatic cancer that combines ultrasound with macrophage depletion, emphasizing the critical role of macrophage dysfunction in tumor immune suppression within the TME. This strategy aims to improve pancreatic cancer treatment by real-time monitoring and precise regulation of tumor-associated macrophage numbers and activity. These studies provide important insights into the construction and reversal of an immunosuppressive TME.

## 3 Application of single-cell sequencing technology in the study of immunity and tumor heterogeneity

With the development of single-cell sequencing technology, scientists can now analyze the heterogeneity of tumors and immune cells at the cellular level, revealing the complex functions of different cell populations in the tumor microenvironment. This technology is of significant importance for understanding the cell-specific mechanisms of immune exhaustion, discovering new therapeutic targets, and is especially crucial in the context of the diverse immune microenvironment in cancer.

In this context, Shao et al. used single-cell RNA sequencing technology to study the subpopulation heterogeneity of malignant epithelial cells in ovarian cancer and found that the high-proliferative C2 TOP2A+ TCs subpopulation was highly sensitive to neoadjuvant chemotherapy (NACT). The study also revealed the critical role of the transcription factor MYBL2 in this subpopulation and developed a prognostic risk model (TTRS) based on this subpopulation to assess the potential impact of tumor heterogeneity on immune therapy. In another article, Li et al. also employed single-cell technology to investigate the immune regulatory role of the TUBA1C gene, revealing its profound impact on the immunosuppressive TME. These studies show that single-cell technologies not only help delineate the specific process of immune cell exhaustion but also unveil how heterogeneity influences cancer immunotherapy resistance and efficacy, providing scientific evidence for personalized treatment strategies.

## 4 Integration of innovative therapeutic approaches and biotechnology

In the context of the continuous advancement of immunotherapy, the combination of nanobiotechnology and imaging-guided technology has opened new possibilities for cancer treatment. By integrating targeted delivery, imaging navigation, and immune modulation, researchers are not only able to enhance the precision of treatments but also regulate the immune microenvironment, laying the foundation for future personalized cancer therapies.

In this field, Xie and Xu demonstrated the potential application of nanotechnology in cervical cancer treatment, particularly by using nanoparticles and stimulus-responsive nanostructures to significantly improve drug and gene delivery efficiency. The study also explored the role of phototherapy, nanogels, and other technologies in enhancing the specificity of drug release and increasing tumor targeting, providing new strategies to overcome resistance issues in traditional therapies. Furthermore, Wang et al. combined ultrasound technology with macrophage depletion strategies to investigate the application of this innovative therapy in pancreatic cancer. Ultrasound guidance not only improved the precision of drug delivery but also enabled real-time monitoring and regulation of immune cell states in the tumor microenvironment, offering new insights into enhancing treatment outcomes and delaying the onset of resistance. These studies represent cutting-edge explorations of nanotechnology and imaging navigation in improving the effectiveness of immunotherapy.

## 5 Conclusion and outlook

The research presented in this Research Topic offers an in-depth exploration of immune cell exhaustion and its complex mechanisms within the tumor microenvironment. The articles provide different perspectives, shedding light on the molecular mechanisms of immune cell dysfunction, the impact of the tumor microenvironment on immune cells, and the potential of innovative technologies and therapeutic strategies to address this issue. Specifically, the studies not only analyze the role of immune-suppressive signaling pathways in cell exhaustion but also demonstrate how single-cell technologies can help uncover the dynamic interactions between tumors and immune cells. Additionally, the research highlights how nanobiotechnology and imaging-guided techniques can enhance the precision and efficacy of immunotherapy.

Despite these important contributions to understanding the mechanisms of immune cell exhaustion and clinical interventions, certain limitations remain. For instance, some studies focus primarily on specific tumor types, and further validation is needed to extend these findings to other cancer types. Furthermore, although several potential therapeutic strategies are proposed, balancing efficacy with side effects and improving treatment sustainability in clinical settings remain challenges that need to be addressed.

As our understanding of the tumor immune microenvironment deepens and technological advancements continue, the prospects for treating immune cell exhaustion are becoming clearer. Future research should focus more on personalized treatment approaches, integrating precision medicine and emerging technologies to provide more targeted solutions for cancer therapy. At the same time, interdisciplinary collaboration and close integration with clinical research will lay the foundation for achieving more effective immunotherapies.
